# Repeated Peritonitis in a Peritoneal Dialysis Patient: An Unusual Case of Bacillus licheniformis With Vancomycin Failure Despite Sensitivity

**DOI:** 10.7759/cureus.65205

**Published:** 2024-07-23

**Authors:** Mohamed Y Ali, Abdalla Fadul, Mohamed O Ali, Mohamed Y Mohamed

**Affiliations:** 1 Internal Medicine, Hamad Medical Corporation, Doha, QAT; 2 Internal Medicine, University of Missouri-Kansas City, Kansas City, USA; 3 Nephrology, Hamad Medical Corporation, Doha, QAT

**Keywords:** vancomycin failure, automated peritoneal dialysis, gram positive bacteria, bacillus licheniformis, peritoneal dialysis related peritonitis

## Abstract

*Bacillus licheniformis *(*B. licheniformis*) is an aerobic, gram-positive, spore-forming rod typically found in soil, decaying organic matter, vegetables, and water, and occasionally part of normal gut flora. This report highlights a case of unusual repeated peritonitis caused by *B. licheniformis*, with three episodes occurring over six months, all of which were sensitive to vancomycin yet presented an unclear cause for recurrence. Peritonitis represents a significant cause of mortality, hospitalization, and failure of peritoneal dialysis catheters, leading to forced transitions to hemodialysis. The rarity of *B. licheniformis* as a pathogen in human infections emphasizes the critical need for precise microbial identification and customized therapeutic strategies.

## Introduction

Peritonitis in peritoneal dialysis (PD) patients remains a predominant cause of mortality, hospitalization, and PD catheter failure with subsequent transitions to hemodialysis modalities [1-3]. While various bacterial pathogens, including *Streptococcus* species, *Staphylococcus aureus *(*S. aureus*), and *Escherichia coli*, have been known as causative agents for peritonitis, atypical pathogens can occasionally emerge and pose diagnostic and therapeutic challenges [4]. This report presents a case of peritoneal peritonitis caused by *Bacillus licheniformis* (*B. licheniformis*), a rarely encountered pathogen in human infections, emphasizing the pivotal role of microbial identification and tailored therapeutic approaches.

## Case presentation

A 55-year-old female with a history of hypertension and end-stage renal disease (ESRD) has been on automated peritoneal dialysis (APD) for five years. The primary cause of kidney disease is unknown. The patient's APD prescription includes an eight-hour duration, 2 L fill volume, 10 L green 2.27%, 1.5 L Extraneal (Baxter Corp., Mississauga, Ontario), and daytime exchange with green 2 liters.

The patient came to the peritoneal dialysis (PD) clinic with a two-day history of abdominal pain and cloudy dialysate. She reported nausea and decreased appetite but no fever, vomiting, or changes in bowel habits. Vital signs were stable on presentation.

Upon clinical examination, the thinly built and fatigued patient exhibited mild tenderness on deep abdominal palpation. Initial laboratory findings showed a WBC of 7.6, with 76% neutrophils. C-reactive protein (CRP) was 7. PD fluid analysis revealed WBC 415, with 61% neutrophils. Laboratory results upon presentation are shown in Table 1.

**Table 1:** Laboratory results upon presentation WBC = white cell count, Hb = hemoglobin, CRP = C-reactive protein, PTH = parathyroid hormone

Lab result	Value	Reference value (unit)
WBC	8.1	4-10 X 10^3 (/ml)
Hb	12.3	12-15 (gm/dl)
Platelets	249	150-450 X 10^3 (/ml)
CRP	7	0-5 (mg/dl)
Urea	9.6	2.5-7.8 (mmol/L)
Creatinine	710	44-80 (mmol/L)
Sodium	137	135-145 (mmol/L)
Potassium	2.7	3.5-5.5 (mmol/L)
Total CO_2_	25	22-29 (mmol/L)
Calcium	2.42	2.2- 2.6 (mmol/L)
Phosphorus	1.59	0.8-1.5 (mmol/L)
PTH	374	15-65 (pg/ml)
Ferritin	529	18-340 (Ug/ml)
Iron saturation	30%	15-45%
Albumin	23	35-50 (Gram/L)

We started empirical treatment with ceftazidime and vancomycin. The PD fluid culture identified *B. licheniformis* as being sensitive to vancomycin. We discontinued ceftazidime, and the patient received vancomycin for three weeks. Symptoms improved, and the total nucleated cell count progressively declined (415 to 159 on day 3). PD fluid cell count post-treatment was 21.

**Figure 1:**
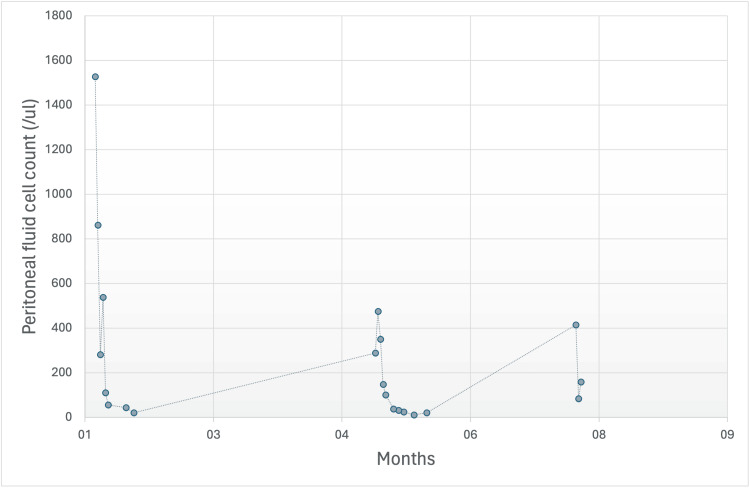
Trend of peritoneal fluid cell count during PD-related peritonitis episodes PD = peritoneal dialysis

The patient had two previous episodes of peritonitis with the same organism, *B. licheniformis*. The first episode presentation was with abdominal pain and cloudy peritoneal fluid for two days. Initial labs showed a WBC count of 9.6 and a CRP of 64. Peritoneal fluid was turbid, with a total nucleated cell count of 1,528 (66% of it is neutrophils), which improved to 281 on days 3 and 21 at the end of the treatment. The trend of cell count during the three presentations is presented in Figure 1.

The second episode was due to abdominal pain. Initial labs showed WBCs of 8.7 and CRP of 3. Peritoneal fluid was not turbid, with a total nucleated cell count of 475, around 60% neutrophils, which improved to 148 on day 3 and to 11 at the end of the treatment. Treatment with vancomycin on both occasions resulted in a positive response.

Further investigation revealed that the patient, a desert tour guide, faced financial challenges that prevented the use of covers during exchanges, which potentially contributed to recurrent peritonitis. Considering repeated episodes and discussions about catheter biofilm, the medical team, in collaboration with vascular surgery, opted to remove the PD catheter. The vascular team inserted a permacath and removed the PD catheter.

The patient transitioned to hemodialysis and is currently doing well with no reported complications.

## Discussion

PD-related peritonitis is a serious complication of PD. It is associated with significant harms, including pain, treatment costs, transfer to hemodialysis and death, and peritoneal adhesions, which can make long-term treatment with PD challenging [5-8]. It is a direct cause of death in >15% of PD patients [5]. International Society for Peritoneal Dialysis (ISPD) recommends monitoring the incidence of peritonitis early and suggests overall peritonitis rates should not exceed 0.40 episodes per year at risk. All PD programs should monitor the incidence of peritonitis as part of a continuous quality improvement (CQI) [9].

Gram-positive organisms other than *S. aureus* and *Staphylococcus epidermidis* account for less than <0.001% [10]. Bacillus species are uncommon causes of PD-related peritonitis, and their incidence is unknown. *B. licheniformis *is an aerobic, gram-positive, spore-forming rod organism usually found in decaying organic matter, soil, vegetables, and water; some species are part of the normal gut flora. It is increasingly recognized as a human pathogen that causes severe infections in debilitated and immunocompromised patients. It was isolated in cases with bacteremia, peritonitis, food poisoning, and eye infection.

Several cases demonstrated it can cause peritonitis, and once detected, it should be treated promptly and not regarded as a contamination. Only three cases have been reported as PD-related peritonitis secondary to *B. licheniformis* [11-13]. Two of these were in continuous ambulatory peritoneal dialysis (CAPD) patients, and one in APD patients. Park et al. reported relapsing *B. licheniformis *peritonitis [12]. It relapsed after treatment with tobramycin and cefazolin but showed a dramatic response after treatment was shifted to vancomycin. All previously reported cases had an excellent response to vancomycin without the need for catheter exchange.

To the best of our knowledge, we present the first case of repeat peritonitis secondary to *B. licheniformis*, with three episodes in six months. In all previous clinical presentations, the isolates were sensitive to vancomycin. In our case, a shared decision between the vascular surgery team and nephrologists was reached to remove the PD catheter and shift temporarily to hemodialysis, as *B. licheniformis* is known to form a biofilm, which can explain the recurrence. The cause of repeat peritonitis is unclear in this case. The PD exchange technique and knowledge were assessed by the nursing team and didn't show significant issues. In previous PD infections, the dose and duration of vancomycin were optimal, and the response was adequate.

To the best of our knowledge, this is the second case of *B. licheniformis* in APD patients. This, and the other case took place in the Arabic peninsula, and two other cases in Korea, which implies that infection risk by this organism might be affected by geographical and environmental factors.

Table 2 compares the four cases of *B. licheniformis* PD catheter peritonitis identified so far.

**Table 2:** Comparison between four cases of reported B. licheniformis PD-related peritonitis DM = diabetes mellitus, HTN = hypertension, CAPD = continuous ambulatory peritoneal dialysis, APD = automated peritoneal dialysis, PD = peritoneal dialysis

	Case 1	Case 2	Case 3	Case 4
Author	Ryoo et al. (2001) [13]	Park et al. (2006) [12]	Albaker (2021) [11]	Current case
Date of publication	2001	2006	2021	Current
Age	56	31	24	55
Sex	Female	Male	Female	Female
Co-morbid condition	DM	HTN	DM	DM
PD modality	CAPD	CAPD	APD	APD
Antibiotic	Netilmicin and cefotiam	Initially, tobramycin and cefazolin, relapsed vancomycin 3 weeks	1 gm of vancomycin 3 weeks	Vancomycin
Previous peritonitis	3	Unknown	0	2
Resolution of peritonitis/outcome	Rapid improvement	Relapse	Rapid improvement	Repeat peritonitis

## Conclusions

*Bacillus licheniformis* is a rare cause of peritoneal dialysis (PD)-related peritonitis. Although previous cases showed an excellent response to vancomycin, our case showed repeat peritonitis. The role of biofilm and the risk of recurrence is not yet well understood. PD exchange knowledge and technique should constantly be revised and reassessed.
